# Prevention of mother-to-child transmission of HIV Option B+ cascade in rural Tanzania: The One Stop Clinic model

**DOI:** 10.1371/journal.pone.0181096

**Published:** 2017-07-12

**Authors:** Anna Gamell, Lameck Bonaventure Luwanda, Aneth Vedastus Kalinjuma, Leila Samson, Alex John Ntamatungiro, Maja Weisser, Winfrid Gingo, Marcel Tanner, Christoph Hatz, Emilio Letang, Manuel Battegay, on behalf of the KIULARCO Study Group

**Affiliations:** 1 Ifakara Health Institute, Ifakara, United Republic of Tanzania; 2 Swiss Tropical and Public Health Institute, Basel, Switzerland; 3 University of Basel, Basel, Switzerland; 4 Saint Francis Referral Hospital, Ifakara, United Republic of Tanzania; 5 ISGlobal, Barcelona Ctr. Int. Health Res. (CRESIB), Hospital Clínic—Universitat de Barcelona, Barcelona, Spain; 6 Division of Infectious Diseases and Hospital Epidemiology, University Hospital Basel, Basel, Switzerland; Hôpital Bichat-Claude Bernard, FRANCE

## Abstract

**Background:**

Strategies to improve the uptake of Prevention of Mother-To-Child Transmission of HIV (PMTCT) are needed. We integrated HIV and maternal, newborn and child health services in a One Stop Clinic to improve the PMTCT cascade in a rural Tanzanian setting.

**Methods:**

The One Stop Clinic of Ifakara offers integral care to HIV-infected pregnant women and their families at one single place and time. All pregnant women and HIV-exposed infants attended during the first year of Option B+ implementation (04/2014-03/2015) were included. PMTCT was assessed at the antenatal clinic (ANC), HIV care and labour ward, and compared with the pre-B+ period. We also characterised HIV-infected pregnant women and evaluated the MTCT rate.

**Results:**

1,579 women attended the ANC. Seven (0.4%) were known to be HIV-infected. Of the remainder, 98.5% (1,548/1,572) were offered an HIV test, 94% (1,456/1,548) accepted and 38 (2.6%) tested HIV-positive. 51 were re-screened for HIV during late pregnancy and one had seroconverted. The HIV prevalence at the ANC was 3.1% (46/1,463). Of the 39 newly diagnosed women, 35 (90%) were linked to care. HIV test was offered to >98% of ANC clients during both the pre- and post-B+ periods. During the post-B+ period, test acceptance (94% versus 90.5%, p<0.0001) and linkage to care (90% versus 26%, p<0.0001) increased. Ten additional women diagnosed outside the ANC were linked to care. 82% (37/45) of these newly-enrolled women started antiretroviral treatment (ART). After a median time of 17 months, 27% (12/45) were lost to follow-up. 79 women under HIV care became pregnant and all received ART. After a median follow-up time of 19 months, 6% (5/79) had been lost. 5,727 women delivered at the hospital, 20% (1,155/5,727) had unknown HIV serostatus. Of these, 30% (345/1,155) were tested for HIV, and 18/345 (5.2%) were HIV-positive. Compared to the pre-B+ period more women were tested during labour (30% versus 2.4%, p<0.0001). During the study, the MTCT rate was 2.2%.

**Conclusions:**

The implementation of Option B+ through an integrated service delivery model resulted in universal HIV testing in the ANC, high rates of linkage to care, and MTCT below the elimination threshold. However, HIV testing in late pregnancy and labour, and retention during early ART need to be improved.

## Introduction

Mother-To-Child Transmission (MTCT) accounts for over 90% of new paediatric HIV infections [1]. The World Health Organization (WHO) has issued several prevention of MTCT (PMTCT) recommendations for low and middle-income countries since 2001 [2,3]. As a result of the scale-up of PMTCT interventions, there has been a 70% decline of new HIV infections among children between 2000 and 2015 [4]. However, in 2015, 23% of HIV-infected pregnant women did not receive effective antiretroviral regimens for PMTCT and 150,000 children acquired HIV [4].

Since 2012, the WHO recommends using lifelong antiretroviral therapy (ART) for all pregnant and breastfeeding women regardless of CD4 counts and clinical stage, and provision of nevirapine or zidovudine to all HIV-exposed infants for four to six weeks regardless of the feeding method. These recommendations are known as “Option B+”.

In Tanzania, Option B+ was rolled out from September 2013 [5]. The MTCT rate in 2014 was 9% [6]. In the Saint Francis Referral Hospital (SFRH) of Ifakara, in rural south-west Tanzania, Option B+ was deployed in April 2014. In 2012, an assessment of the PMTCT circuit had identified several gaps, namely: (a) poor linkage into HIV care of newly diagnosed HIV-infected pregnant women; (b) no re-testing of seronegative women in late pregnancy; and (c) lack of a standardised follow-up of HIV-exposed infants [7]. To bridge these gaps, an integrated and comprehensive service delivery model to improve maternal and paediatric HIV care was implemented in parallel to Option B+ [8,9].

The current study describes the PMTCT cascade and uptake of Option B+ guidelines implemented through this service delivery model.

## Methods

This is a prospective cohort study describing the PMTCT cascade and uptake of Option B+ guidelines in the SFRH. The uptake of PMTCT recommendations was compared with the one previously described in 2012 [7]. We also assessed the MTCT rate, characterised HIV-infected mothers and analyzed the differences between newly diagnosed pregnant women and women who became pregnant while being under HIV care.

### Study setting and population

The SFRH is the largest healthcare facility in the rural Kilombero district, serving a population of 600,000 inhabitants and an estimated 30,000 people living with HIV [10]. HIV care and treatment is offered within the hospital at the Chronic Diseases Clinic of Ifakara. The clinic works in collaboration with the Ifakara Health Institute, the Swiss Tropical and Public Health Institute and the Department of Infectious Diseases and Hospital Epidemiology of the University Hospitals of Basel and Bern, Switzerland. Since 2004, all patients attending the clinic are invited to participate in the Kilombero and Ulanga Antiretroviral Cohort (KIULARCO) [11–14]. Written informed consent is sought from all participants; for children and adolescents aged < 18 years, informed consent is sought from caregivers. The KIULARCO study obtained ethical approval from the Ifakara Health Institute ethical review board, the National Institute for Medical Research of Tanzania, the Tanzanian Commission for Science and Technology and the ethical review board of Northwest Switzerland. It is the largest rural HIV cohort in Tanzania, with over 9,000 patients ever enrolled. Patients receive care according to the National AIDS Control Program [15].

**Fig 1:**
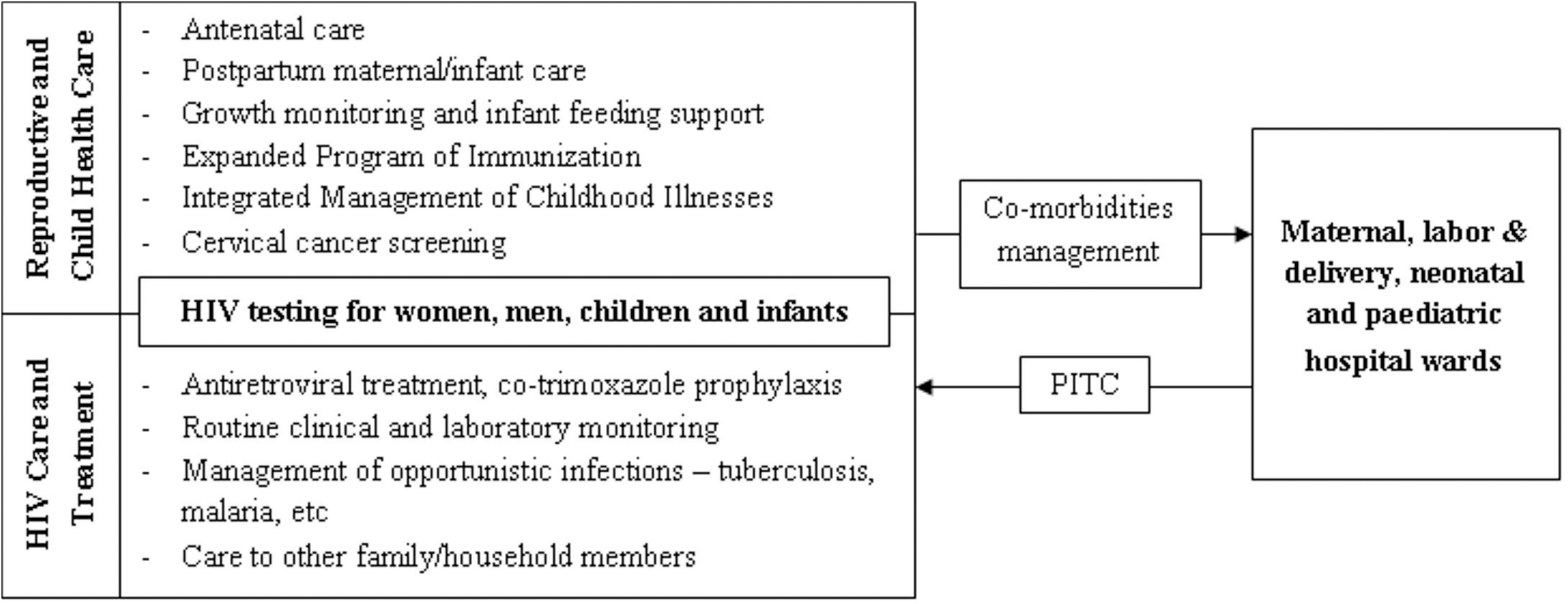
The One Stop Clinic of Ifakara integrates health services for HIV-infected pregnant women, children, HIV-exposed infants and their families in a rural Tanzanian hospital. PITC: Provider Initiated HIV Testing and Counseling.

In December 2012 the One Stop Clinic of Ifakara was created, a maternal and paediatric family-oriented HIV unit integrated within the Reproductive and Child Health Clinic. The services delivered include: antenatal care (ANC), postpartum maternal and infant care, cervical cancer screening, child’s growth and health monitoring, expanded program of immunizations, nutritional assessment and tuberculosis and HIV care and treatment (Fig 1). Details about the organization, functioning and staff of the One Stop Clinic have been published elsewhere [8,14].

In this study we included all pregnant women and HIV-exposed infants attended in the ANC station, labour ward and/or the Chronic Diseases Clinic of Ifakara in SFRH during the first year of Option B+ implementation, from April 1^st^, 2014 to March 31^st^, 2015. Data from HIV-infected pregnant women was analysed by February 11^th^, 2016. For HIV-exposed infants, given the long breastfeeding period, retention in care and final HIV serostatus was analysed as per June 30^th^, 2016. HIV-infected pregnant women transferred to the One Stop Clinic from other facilities were excluded. Lost to follow-up was defined as not having visited the clinic 60 days after the last scheduled date for HIV-infected mothers and 90 days for HIV-exposed infants.

### Statistical analysis

Characteristics of pregnant women were summarised using proportions and medians. The comparison of data from ANC and labour ward during the pre-B+ and post-B+ periods was done using proportion differences. Comparisons between newly enrolled pregnant women and women who became pregnant while being under care were made using Chi-square and Wilcoxon rank-sum tests for categorical and continuous variables, respectively. Fisher exact test was used to compare proportions of women’s follow-up status. Finally, we described the characteristics of the HIV-exposed infants enrolled and reported the MTCT rate. SAS 9.3 was used for data analysis (SAS Institute Inc., Cary, NC, USA).

## Results

### Implementation of a new PMTCT care pathway

**Fig 2:**
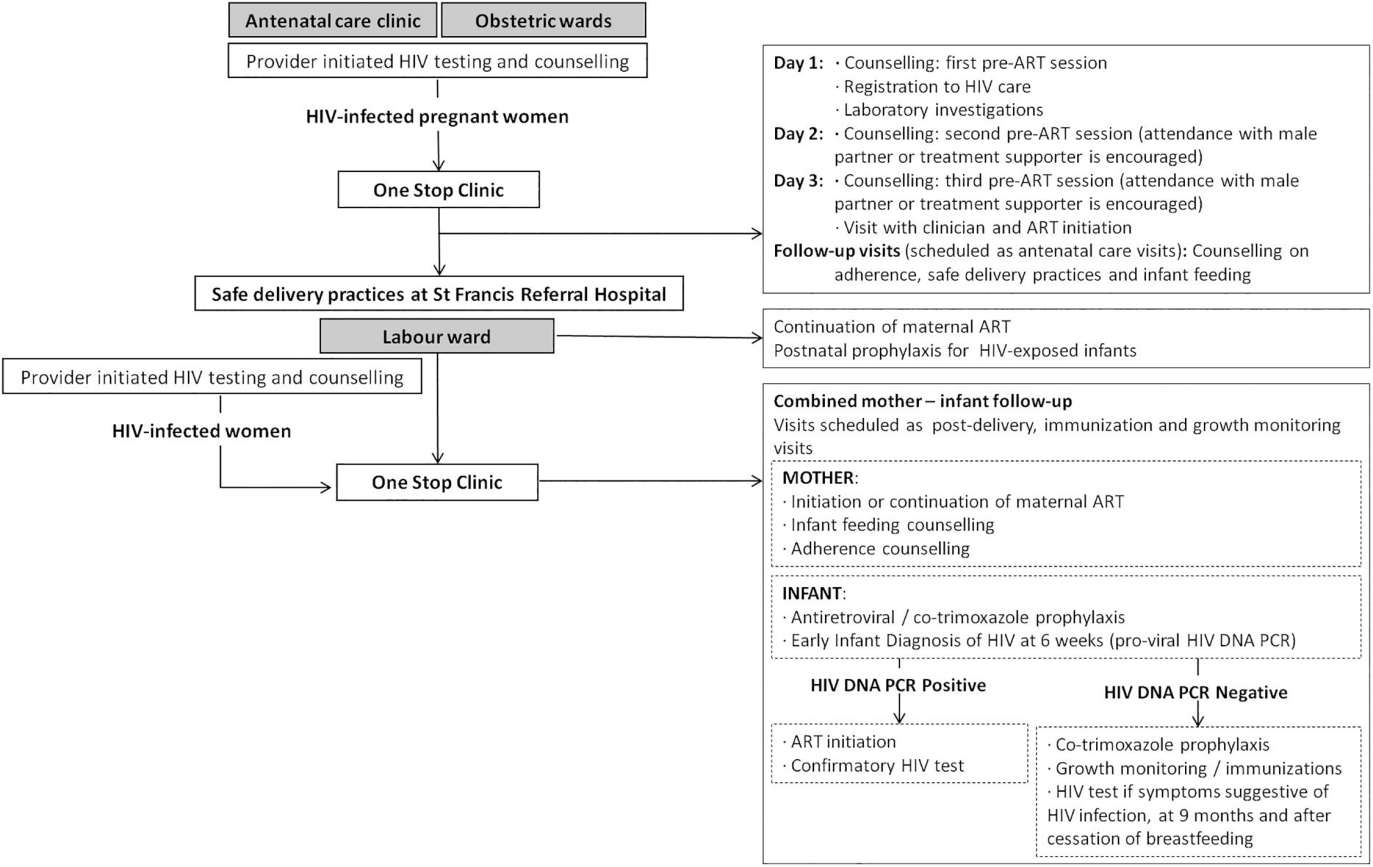
Current prevention of mother-to-child transmission of HIV care pathway at Saint Francis Referral Hospital. ART: antiretroviral treatment.

Since 2013 comprehensive PMTCT services are delivered within the Reproductive and Child Health Clinic [3,5] and coordinated by the One Stop Clinic. These services involve different hospital units: the ANC, the HIV clinic and the labour ward. Option B+ services include: 1) routine HIV counselling and testing for pregnant women and male partners; 2) comprehensive antenatal care; 3) lifelong ART for HIV-positive pregnant and breastfeeding women; 4) safe delivery practices; 5) postpartum care for mothers and infants; 6) antiretroviral and co-trimoxazole prophylaxis for HIV-exposed infants; 7) counselling for safe infant feeding practices; 8) early infant HIV diagnosis and treatment for HIV-infected infants; 9) male partner and family involvement. Fig 2 illustrates the PMTCT care pathway at SFRH.

### PMTCT at the antenatal clinic

From April 1^st^, 2014 to March 31^st^, 2015, 1,579 women attended the ANC and 0.44% (7/1,579) were previously known to be HIV-infected. Ninety-eight percent of women with unknown HIV serostatus (1,548/1,572) were offered an HIV test and 94% (1,456/1,548) accepted. Thirty-eight (38/1,456; 2.6%) were found to be HIV-positive. Among the seronegative women, 51/1,418 (3.6%) were re-tested for HIV during late pregnancy and one (2%) had seroconverted. In total, 46 women were identified as HIV-infected in the ANC. The overall HIV prevalence at the ANC was 3.1% (46/1,463). Compared to the period 01/2010-12/2011, acceptance of HIV testing improved (94% versus 90.5%) after Option B+ implementation (p<0.0001) and the HIV prevalence at the ANC decreased from 6.9% to 3.1% (p<0.0001; Table 1).

**Table 1:** Changes in the uptake of the prevention of mother-to-child transmission of HIV recommendations before and after Option B+ implementation.

PMTCT step	Description		Pre-Option B+ period	Post-Option B+ period	p-value
**Assessment of HIV status in antenatal care**			**01/01/2010–31/12/2011**	**01/04/2014–31/03/2015**	
	Number of women attended at the ANC		4027	1579	
	Pregnant women with HIV positive status known, n/N (%)		44/4027 (1.1)	7/1579 (0.4)	0.0213
	Pregnant women offered an HIV test, n/N (%)		3983/3983 (100)	1548/1572 (98.5)	0.0107
	Pregnant women accepting the HIV test, n/N (%)		3606/3983 (90.5)	1456/1548 (94.1)	<0.0001
	Pregnant women tested HIV positive at the ANC, n/N (%)		207/3606 (5.7)	39/1456 (2.7)	<0.0001
	Prevalence of HIV among pregnant women attended at the ANC, n/N		251/3650 (6.9)	46/1463 (3.1)	<0.0001
**Enrolment in HIV care**			**01/01/2010–31/12/2011**	**01/04/2014–31/03/2015**	
	Women newly diagnosed at ANC and enrolled, n/N (%)		53/207 (25.6)	35/39 (89.7)	<0.0001
**PMTCT at labour ward**			**20/03/2011–03/05/2011**	**01/04/2014–31/03/2015**	
	Number of deliveries		570	5727	
	Women with HIV positive status known at admission, n/N (%)		28/570 (4.9)	168/5727 (2.9)	0.01
	Women with unknown HIV status at admission, n/N (%)		82/570 (14.4)	1155/5727 (20.2)	<0.0001
		Women tested for HIV during labour & delivery, n/N (%)	2/82 (2.4)	345/1155 (29.9)	<0.0001
		Women tested HIV-positive during labour & delivery, n/N (%)	1/2 (50)	18/345 (5.2)	
	Prevalence of HIV among women attended at labour ward, n/N (%)		29/486 (6.0)	186/4917 (3.8)	0.0188
	HIV-exposed newborns receiving the recommended postnatal prophylaxis, n/N (%)		1/29 (3.5)	154/184 (83.7)	<0.0001

PMTCT: Prevention of mother-to-child transmission of HIV; ANC: antenatal care clinic

### Pregnant women receiving HIV care at the One Stop Clinic

During the study period, 124 HIV-infected pregnant women attended the One Stop Clinic. Forty-five of them had been newly diagnosed with HIV during the current pregnancy, and 79 were under HIV care when pregnancy was reported.

#### Newly diagnosed HIV-infected pregnant women

Of the 39 pregnant women newly diagnosed in the ANC, 35 (90%) were enrolled into HIV care, a significant increase from the period 01/2010-12/2011 (90% versus 26%, p<0.0001) (Table 1). Another ten women were newly diagnosed and enrolled in HIV care during the study period: 7/10 from the obstetric ward and 3/10 from the voluntary counselling and testing unit. The clinical characteristics and pregnancy outcomes of these 45 women are summarized in Table 2. Median age at enrolment was 28.4 years (interquartile range (IQR) 22.2–31.8), median CD4 counts 334 cells/μL (IQR 166–509), and 89% presented with WHO stage 1 or 2. Seventeen women (38%) were aware of their male partner HIV serostatus: 7/45 (16%) reported their male partner was HIV-positive and 10/45 (22%) HIV-negative. Thirty-seven women (82%) initiated ART after a median of 3 days (IQR 0–7). After a median follow-up time of 17.2 months (IQR 14.8–21.2), 28/45 (63%) women were under active follow-up at the One Stop Clinic, 4/45 (9%) had been transferred to another facility, 1/45 (2%) had died and 12/45 (27%) were lost to follow-up. Pregnancy outcomes were recorded for 35/45 women: 6/35 (17%) had an abortion, miscarriage or neonatal death, and the remainder delivered alive babies.

**Table 2:** Characteristics of HIV-infected women enrolled in HIV care during pregnancy and women enrolled before pregnancy.

Characteristics				New HIV diagnosis during pregnancy (N = 45)	Women under HIV care who became pregnant during follow-up (N = 79)	p-value
**Time since enrolment (months), median (IQR)**				-	55 (15–74)	
**Age at pregnancy report (years), median (IQR)**				28.4 (22.2–31.8)	34 (29–36)	<0.0001
**CD4 count at enrolment (cells/μL), median (IQR)**				334 (166–509)	265 (134–495)	0.46
**WHO clinical stage at enrolment, n (%)**						0.05
	Stage 1 or 2			40 (88.9)	59 (78.7)	
	Stage 3 or 4			5 (11.1)	16 (21.3)	
**Partner HIV serostatus at pregnancy report, n (%)**						0.02
	Positive			7 (15.6)	31 (39.2)	
	Negative			10 (22.2)	16 (20.3)	
	Not tested / Unknown			21 (46.7)	19 (24.1)	
	Not applicable ^a^			7 (15.6)	13 (16.5)	
**ART status at pregnancy report**						
	Not on ART, n (%)			-	11 (13.9)	
	On ART, n (%)			-	68 (86.1)	
		Time on ART at pregnancy report (months), median (IQR)		-	45.3 (16.3–72.1)	
		ART regimen at pregnancy report, n (%)				
			NVP-based regimen	-	19 (27.9)	
			EFV-based regimen	-	41 (60.3)	
			PI-based regimen	-	8 (11.8)	
**ART-naïve pregnant women initiated on ART, n/N (%)**				37/45 (82.2)	11/11 (100)	
**Time to ART initiation after pregnancy report (days), median (IQR) ^b^**				3 (0–7)	2 (0–5)	
**Follow-up time at time of analysis (months), median (IQR)**				17.2 (14.8–21.2)	19.1 (14.5–21.8)	
**Retention in care at time of analysis, n (%)**						0.02
	Active follow-up			28 (62.2)	64 (81.0)	
	Died			1 (2.2)	3 (3.8)	
	Lost to follow-up			12 (26.7)	5 (6.3)	
	Transfer to another clinic			4 (8.9)	7 (8.9)	
**Pregnancy outcome ^c^, n (%)**						0.05
	Live born singleton			27 (77.1)	64 (85.3)	
	Live born twins			2 (5.7)	2 (2.7)	
	Abortion, miscarriage or neonatal death			9 (12.0)	6 (17.1)	

^a^ Not applicable refers to women who denied having a male partner

^b^ Applies to 37/45 (N = 37) of the newly diagnosed women and to women under HIV care who were not on ART at pregnancy report (N = 11)

^c^ For 10 newly diagnosed women and 4 women under HIV care at pregnancy report, pregnancy outcome was nor recorded (they died, were lost to follow-up or were transferred to another clinic before delivery)

IQR: interquartile range; WHO: World Health Organization; ART: antiretroviral treatment; NVP: nevirapine; EFV: efavirenz; PI: protease inhibitor.

#### Women under HIV care who became pregnant

Seventy-nine women under HIV care became pregnant during the study period. The median time since enrolment was 55 months (IQR 15–74). At the time of HIV diagnosis, their median CD4 counts were 265 cells/μL (IQR 134–495) and 79% presented WHO stage 1 or 2 (Table 2). When pregnancy was reported, 47/79 (60%) were aware of their male partner’s serostatus: 31/79 (39%) reported their male partner was HIV-positive and 16/70 (20%) HIV-negative. Most women (68/79, 86%) had started ART before pregnancy. The remaining 11 women initiated ART after a median of 2 days of pregnancy report (IQR 0–5). After a median follow-up of 19.1 months (IQR 14.5–21.8), 64/79 (81%) women were under active follow-up at the One Stop Clinic, 7/79 (9%) had been transferred to another facility, 3/79 (4%) had died and 5/79 (6%) were lost to follow-up. Pregnancy outcomes were recorded for 75/79 women: 9/75 (12%) had an abortion, miscarriage or neonatal death and the rest delivered alive babies (Table 2).

**Fig 3:**
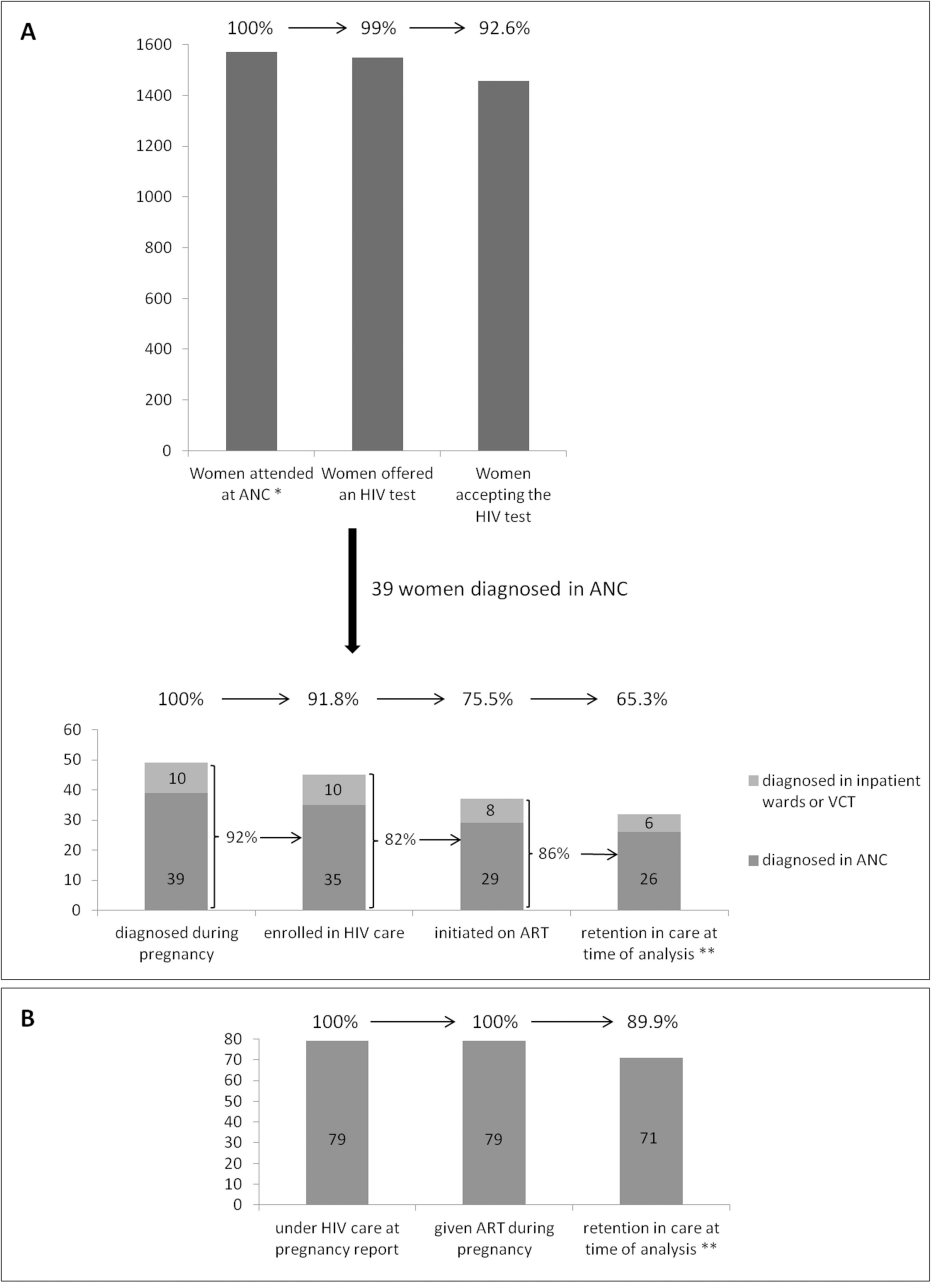
Retention through the PMTCT cascade for women newly diagnosed with HIV during the current pregnancy (3A) and women who became pregnant while being under HIV care (3B). * Women known to be HIV-infected (n = 7) have not been included. ** Women retained in care at the One Stop Clinic and women transferred to another facility were considered to be under active follow-up. PMTCT: prevention of mother-to-child transmission of HIV; ANC: antenatal care clinic; ART: antiretroviral treatment; VCT: voluntary HIV counselling and testing.

Fig 3 shows the PMTCT cascade for newly diagnosed women (3A) and women becoming pregnant while under HIV care (3B). Compared to newly diagnosed women, those who were under HIV care at pregnancy report were older (34 versus 28.4 years, p<0.0001), had similar CD4 counts at HIV diagnosis (265 versus 334, p = 0.46), higher proportion of WHO clinical stage 3 or 4 (21% versus 11%, p = 0.05), better knowledge of their male partner serostatus (60% versus 38%, p = 0.02), lower lost to follow-up rate (6% versus 27%, p = 0.02), and better pregnancy outcomes (proportion of live born babies 88% versus 83%, p = 0.05) (Table 2).

### PMTCT at the labour ward

The labour ward at SFRH attends women from several ANCs and receives the referrals from lower level facilities. During the study period 5,727 women delivered at the hospital. At admission, 80% (4,572/5,727) had their HIV status documented (168 HIV-positive) and for 20% (1,155/5,727) it was unknown. Thirty percent (345/1,155) of women in this latest group were tested for HIV during labour, 5.2% (18/345) were HIV-infected. Overall, 3.8% (186/4,917) of women with known HIV serostatus giving birth at SFRH were HIV-infected. None of the women with a documented HIV-negative test during pregnancy was re-tested during labour. The comparison of the uptake of recommendations between our previous assessment and the current period is presented in Table 1. During the post-B+ period the number of women presenting at labour with unknown HIV status increased (20% versus 14%, p<0.0001), but the HIV testing rate of these women increased (30% versus 2.4%, p<0.0001). Similarly to the ANC, the HIV prevalence among pregnant women attended at the labour ward was lower than in 2010–11 (3.8% versus 6.0%, p = 0.02).

### HIV-exposed infants and mother-to-child transmission

During the study period, 135 HIV-exposed infants born from mothers diagnosed with HIV before pregnancy or during pregnancy or delivery were enrolled at the One Stop Clinic (Table 3). Sixty-two percent (83/135) of the mothers were diagnosed before pregnancy. Eighty eight percent (119/135) of the mothers received correct drugs for PMTCT and 75% (101/135) of the infants got the recommended postnatal prophylaxis. Median age at the time of the first HIV test was 6 weeks (IQR 5–11). By the time of analysis 2.2% (3/135) infants were HIV-infected (all diagnosed at first HIV test), 66.7% (90/135) were uninfected, and 31.1% (42/135) had a first negative HIV test but were still breastfeeding when last visited. Remarkably, none of the followed infants with an initial negative test seroconverted during the study period. Fifteen months after the last infant included was enrolled, the lost to follow-up rate was 14% (19/135) and 6% (8/136) of the infants had died (2 were HIV-infected).

**Table 3:** Characteristics of the 135 HIV-exposed infants born from mothers diagnosed before pregnancy or during pregnancy, labour and delivery.

Characteristic			
**Female gender, n (%)**			63 (46.7)
**Time of mother’s HIV diagnosis, n (%)**			
	Before pregnancy		83 (61.5)
	During pregnancy/delivery		52 (38.5)
**Correctness of mother’s ART for PMTCT, n (%)**			
	Correct		119 (88.2)
	Incorrect		16 (11.9)
**Correctness of infant’s postnatal ARV prophylaxis, n (%)**			
	Correct		101 (74.8)
	Incorrect		34 (25.2)
**Age at first HIV test (weeks), median (IQR)**			6 (5–11)
**Timely initiation of co-trimoxazole prophylaxis, n (%) ^a^**			108 (84.4)
**Infant feeding during the first 6 months of life, n (%)**			
	Exclusive breastfeeding		123 (91.1)
	Replacement feeding		4 (3.0)
	Animal’s milk		2 (1.5)
	Mixed feeding		6 (4.4)
**Final HIV serostatus, n (%)**			
	HIV negative		90 (66.7)
	HIV positive		3 (2.2)
	Not yet known ^b^		42 (31.1)
**Retention in care at time of analysis, n (%)**			
	Active follow up		103 (76.3)
	Lost to follow-up		19 (14.1)
	Transferred to another clinic		5 (3.7)
	Died ^c^		8 (5.9)
		HIV-positive	2

^a^ For 7 infants the correctness of CPT initiation was not applicable, since they were enrolled after the age of 6 weeks

^b^ 19/42 infants were lost to follow-up after a first negative HIV test; 23/42 infants had a first negative HIV test but were still breastfeeding at the time of analysis

^c^ Causes of death: 1 septicaemia (HIV-infected); 1 acute respiratory failure (HIV-infected); 1 congenital hydrocephaly; 1 spina bifida; 1 Kwashiorkor malnutrition (and suspected tuberculosis); 1 bacterial pneumonia; 2 unknown.

ART: antiretroviral treatment; PMTCT: prevention of mother-to-child transmission of HIV; ARV: antiretroviral; IQR: interquartile range.

During the same period 24 HIV-exposed infants born from mothers who were diagnosed after delivery were enrolled. Most of these mothers had attended an ANC during pregnancy but were not offered an HIV test. The MTCT rate in this group was 46% (11/24). Most of these infants and their mothers were identified in the hospital wards, when admitted with malnutrition, advanced HIV disease or opportunistic infections.

## Discussion

This is the first study to evaluate the Option B+ cascade in Tanzania. Option B+ delivered through the One Stop Clinic model dramatically improved linkage to HIV care after diagnosis in the ANC and resulted in over 90% of enrolled women receiving ART. Retention throughout the PMTCT pathway was challenging for newly diagnosed HIV-infected pregnant women. The observed MTCT rate (2.2%) was below the national average (9%) and the threshold established for elimination of MTCT of HIV in breastfeeding populations (5%) [16]. Nevertheless, gaps such as the poor uptake of HIV testing in the labour ward and an almost inexistent HIV re-screening during late pregnancy remained and need to be urgently addressed.

### Improvement in enrolment to HIV care

The rate of HIV testing in the ANC was above 90% both in the pre-B+ and the post-B+ periods. This is reassuring, since failure to detect HIV-infected mothers in the ANC is estimated to be responsible for 54% of paediatric HIV infections in resource-limited settings [17]. Enrolment to HIV care from ANC increased from 26% during 01/2010-12/2011 to 90% during 04/2014-03/2015. In two large urban centres in Malawi, the enrolment to PMTCT/HIV care from the ANC increased from 61% in the pre-B+ period to 87% in the post-B+ period [18]. However, a study from Uganda found that after Option B+ implementation, only 25% of women diagnosed in rural ANC settings were linked to HIV care. Health workers interviewed in that study suggested improving competence in HIV counselling and integration of PMTCT and chronic HIV care within the routine reproductive and child care as potential solutions to poor linkage [19]. Thus, we attribute the improved linkage to HIV care, not to Option B+ itself, but to the integration of antenatal and HIV services within the same clinic, the good coordination between both, and the specialised counselling delivered through the One Stop Clinic.

### The main challenge: Retention through the PMTCT cascade

In our study, Option B+ was challenged by the losses of newly diagnosed HIV-infected women along the PMTCT cascade. As seen in other settings [18], a substantial proportion of newly enrolled women (8/45, 18%), were lost to follow-up before ART initiation. High rates of early attrition are also seen in places where same day diagnosis and treatment initiation is the standard of care [20,21]. These findings indicate that women may need time to adjust to the HIV diagnosis and understand the benefits of lifelong ART. After ART initiation, recently diagnosed women continued to drop from care, although at a lower rate: 86.5% (32/37) were retained after a median of 17.2 months. This result is better than the one reported in a recent publication from Malawi, where retention after ART initiation in the context of Option B+ was 68.5% at 12 months, 61% at 24 months and 56.3% at 36 months [22]. While the size of our study population was small, our better results suggest that the unhurried start of ART and continued counselling combined with joint visits for maternal care, PMTCT/HIV care and infant growth monitoring and immunization may be responsible for the lower attrition. The better retention of women becoming pregnant while being under HIV care must be capitalized and peer-mother programs should be common in all settings [23,24]. Importantly, the uniqueness of the challenges of Option B+ may be coming to an end, since many countries are implementing the “test and treat” strategy for all HIV-infected individuals [25]. Thus, lessons learned from the implementation of Option B+ should serve to enhance not only PMTCT but also ART programs in sub-Saharan Africa.

### The elimination of new paediatric HIV infections: A met target

The MTCT rate observed at the One Stop Clinic was 2.2%, achieving the target of <5% established for populations in which breastfeeding is common [16]. Therefore, unlike previously reported [26], the virtual elimination of new paediatric HIV infections is feasible in a rural setting under programmatic circumstances. The lost to follow-up rate of infants after a minimum of 15 months since enrolment was 14%, much lower than the average 34% reported for sub-Saharan African settings at only 3 months post-delivery [27]. We believe that the integrated service delivery model combined with continued counselling after delivery are responsible for these results. In line with our findings, a cluster-randomized clinical trial conducted in Nigeria showed that integration of mother and infant services resulted in better provision of PMTCT and a significant increase in retention [28].

This success is shadowed by the infants identified as HIV-exposed after delivery. In most cases, these infants were enrolled after being admitted with symptoms and a high proportion was HIV-infected. This finding provides evidence that some mothers are still not captured by PMTCT programs. Thus, in parallel to Option B+ efforts, case-finding approaches are necessary to timely diagnose HIV-infected children outside the PMTCT pathway [29].

### Testing gaps

Twenty percent of women attended the labour ward with unknown HIV serostatus. Since over 95% of women in the region attend at least one antenatal visit [30], we attribute this finding to test shortages in some district health facilities. Intrapartum HIV testing is crucial for maximizing PMTCT programs, but, worryingly, only a third of women with unknown serostatus were tested during labour. Reasons for the low HIV testing at the labour ward are the substantial workload of the health workers and the frequent staff turnover, which hinder the efficacy of the PMTCT training and messages delivered to midwifes and nurses working there. Furthermore, HIV re-screening during late pregnancy was anecdotic in our setting. A second HIV test during the last trimester of pregnancy is recommended based on its cost-effectiveness [31] and the increased risk of HIV acquisition during pregnancy [32]. In our hospital, 2% of women re-tested for HIV in late pregnancy had seroconverted. With this seroconversion rate, a total of 28 women may have been silently infected during pregnancy. Having re-screened all women would have resulted in a 72% increase in the number of women diagnosed in the ANC and an opportunity to prevent their infants to become infected. Re-screening and intrapartum testing can act as safety nets to ensure the success of PMTCT. Continuous education of the attending staff and a strengthen supply chain of tests are crucial to bridge these testing gaps.

This study has some limitations. First, we did not include mother-infant pairs, but pregnant women and HIV-exposed infants enrolled during the study period. The number of infants enrolled in a year exceeded the number of women. This is due to yearly variations and transferred and orphan infants, but also to some mothers from peripheral facilities preferring their infants to receive comprehensive care at the One Stop Clinic. Still, a significant proportion of the infants were born from mothers enrolled during the study, and the uptake of the guidelines could be satisfactorily assessed. Second, an important part of women delivering at SFRH did not attend the local ANC, and complete antenatal data was not available for them. Third, it is possible that some women ascertained as lost to follow-up self-transferred to another facility, as it is common in district high level facilities as ours [33,34]. Finally, since 14% of the HIV-exposed infants were lost to follow-up while breastfeeding, it is possible that the MTCT rate of 2.2% underestimates the true transmission rate. However, even presuming a 15% transmission through the breast milk among the lost to follow-up infants, the cohort MTCT rate would still be below 5%.

The strength of this study is being the first one to comprehensively analyse the Option B+ cascade in a Tanzanian setting, including all the steps of the PMTCT pathway and the long follow-up time for both mothers and infants.

## Conclusions

In summary, Option B+ was successfully implemented in this rural African setting through an integrated service delivery model. Most diagnosed women were linked into HIV care, received appropriate ART and the MTCT rate was below 5%. However, important testing gaps that may have left women undiagnosed were observed. The One Stop Clinic is a feasible, inexpensive and scalable Option B+ delivery model that could be extrapolated to similar rural settings. Despite the success, caution is warranted and additional strategies to ensure universal HIV testing for pregnant and delivering women and to improve early ART retention of newly diagnosed women are crucially needed.

## Supporting information

S1 File Minimal dataset. (XLSX)
